# Clinical efficacy and neurobiological correlates of electroconvulsive therapy in patients with clozapine-resistant/intolerant schizophrenia: study protocol of multi-site parallel arm double-blind randomized sham-controlled study

**DOI:** 10.12688/wellcomeopenres.18028.2

**Published:** 2022-10-05

**Authors:** Shyam Sundar Arumugham, Samir K. Praharaj, Umesh Shreekantiah, Vanteemar S. Sreeraj, Chandramouli Roy, Sonia Shenoy, Abhiram Narasimhan Purohith, Uppinkudru Chithra, Kiran Basawaraj Bagali, Sudhir Venkataramaiah, Gopala Krishna Kadarapura Nanjundaiah, Kandavel Thennarasu, Channaveerachari Naveen Kumar, Nishant Goyal, Basudeb Das, Urvakhsh Meherwan Mehta, Kesavan Muralidharan, Ganesan Venkatasubramanian, Preeti Sinha, Jagadisha Thirthalli

**Affiliations:** 1 Department of Psychiatry, National Institute of Mental Health and Neurosciences, India, Bengaluru, Karnataka, India, 560029, India; 2 Department of Psychiatry, Kasturba Medical College, Manipal, Manipal Academy of Higher Education, Manipal, Karnataka, 576104, India; 3 Central Institute of Psychiatry, Ranchi, Jharkhand, 834006, India; 4 Department of Neuroanaesthsia and Neuro Critical Care, National Institute of Mental Health and Neurosciences, India, Bengaluru, Karnataka, 560029, India; 5 Department of Biostatistics, National Institute of Mental Health and Neurosciences, India, Bengaluru, Karnataka, 560029, India

**Keywords:** electroconvulsive therapy, clozapine, treatment-resistance, schizophrenia

## Abstract

**Background:** A substantial proportion of patients with treatment resistant schizophrenia do not respond well or partially to clozapine, with a subset that does not tolerate an adequate trial of clozapine. Electroconvulsive therapy (ECT) is regarded as one of the augmenting options, but there is a lack of high-quality evidence for this practice. This protocol describes a double-blind randomised sham-controlled modified-ECT trial to evaluate its efficacy in patients with clozapine resistant/intolerant schizophrenia. The study also involves multimodal investigations to identify the response predictors and the mechanistic basis of modified ECT in this population.

**Methods:** One hundred consenting schizophrenia patients with resistance/intolerance to clozapine referred by clinicians for ECT would be randomly assigned to receive true ECT or sham ECT at three study centers. Sham ECT would mimic all the procedures of modified ECT including anaesthesia and muscle relaxation, except the electrical stimulation. After a blinded course, non-responders to sham ECT would be offered open-label true ECT. Clinical assessments, neurocognitive assessments and multimodal investigations (magnetic resonance imaging [MRI], electroencephalography, heart rate variability, investigative transcranial magnetic stimulation-transcranial direct current stimulation, gene polymorphism) would be conducted at baseline and repeated after the end of the trial, as well as open-label ECT course. The trial would evaluate the improvement in positive symptoms (scale for assessment of positive symptoms) of schizophrenia as the primary outcome measure with prediction of this change by resting-state functional-MRI based brain-connectivity as the second primary objective.

**Registration:** Clinical Trial Registry of India (Reg no: CTRI/2021/05/033775) on 24 ^th^ May 2021.

## Background and rationale

Clozapine-resistant schizophrenia remains a major challenge in the practice of psychiatry with limited evidence-based interventions ( Remington *et al.*, 2017). Most people with this condition, the majority of whom are relatively young, lead their lives with substantial subjective distress and disability. Electroconvulsive therapy (ECT) is sometimes recommended by clinical practice guidelines ( Kane *et al.*, 2019; Taylor *et al.*, 2021) and is commonly prescribed for this condition.

Unlike in major depressive disorder, the quality of evidence to support the efficacy of ECT in schizophrenia is limited. The gold standard evidence for the effectiveness of any intervention is a double-blind randomized controlled trial (RCT) ( Hariton & Locascio, 2018), with a placebo comparator arm that mimics all aspects of the intervention except the active ingredient. The ideal comparator arm for ECT would be a sham ECT ( Ross, 2006), that mimic modified-ECT (including anaesthesia and muscle relaxant) without an electrical stimulus. There have been earlier sham-controlled ECT trials in schizophrenia, which have evaluated the efficacy of ECT predominantly as a combination treatment with antipsychotics as a first-line treatment. In a Cochrane review of 12 such studies by Tharyan & Adams (2005), six studies found ECT to be more effective, while in the remaining six studies, the results were equivocal. The results were not meta-analysed, and hence the superiority of antipsychotic medication plus ECT over the continuation of antipsychotics alone (plus no ECT/sham ECT) cannot be established with this review. Furthermore, none of these studies were conducted in a population with clozapine resistance or those that did not tolerate clozapine. 

To date, the best available evidence for ECT in a clozapine-resistant population is a randomized, single-blind comparison with clozapine alone, which has shown promising results for the ECT group, with around 50% of the patients responding to ECT ( Petrides *et al.*, 2015). However, the improvement in the ECT group in this non-blinded study cannot be attributed to the direct therapeutic effect of ECT alone. A recent meta-analysis of 18 such RCTs comparing ECT augmentation of clozapine with clozapine alone found a statistically significant improvement in the former group compared to the latter ( Wang *et al.*, 2018). None of these had a sham comparator arm, thus compromising the blinding of participants. The quality of the RCTs was also questionable, with suboptimal methods or inadequate information on randomisation, allocation concealment, and rater blinding. Most of these trials were not registered, thereby allowing a potential for selective reporting. 

Furthermore, it is notable that studies have shown a high response to sham ECT too. For example, studies on major depressive disorder have shown that around 30–50% of patients improve with sham ECT alone ( Rasmussen, 2009). In treatment-resistant schizophrenia, a reduction of around 20% in Brief Psychiatric Rating Scale scores has been observed with sham ECT ( Goswami *et al.*, 2003), which is a commonly employed cut-off for treatment response in this population ( Kane *et al.*, 1988). This improvement can be attributed to other factors, including placebo response, concomitant treatment, anaesthetic agents, and the natural course of illness ( Fox, 2010; Rasmussen, 2009). Moreover, the positive effects of care that are as intensive as surgical intervention, the use of technological intervention, attention provided by the medical staff, and prior belief in the effectiveness of the treatment also add to the placebo effect of ECT. As in depressive disorder, only a sham-controlled trial would conclusively prove the efficacy of ECT in this population. The only published sham-controlled ECT trial in this population found no significant difference in Positive and Negative Syndrome Scale (PANSS) scores between the true and sham groups ( Melzer-Ribeiro *et al.*, 2017). However, given the small sample size (n=23), the study might have been underpowered to detect statistically significant differences. There is evidence that some forms of ECT are more efficacious than others in schizophrenia ( Phutane *et al.*, 2011). One may consider this to be equivalent of evidence from sham-ECT controlled studies, as, in these studies, the less effective form of ECT may be considered as equivalent of sham-ECT. However, these studies included mixed groups of patients with schizophrenia; the proportion of patients with clozapine resistant schizophrenia in these studies is unknown. Hence, there is no conclusive evidence to support the efficacy of ECT in clozapine-resistant schizophrenia. In the context of poor-quality evidence supporting the use of ECT in treatment resistant schizophrenia, the report of the Royal College of Psychiatrists’ Special Committee on ECT states that *“Claims for the efficacy of ECT in treatment-resistant schizophrenia would perhaps best be described as a triumph of anecdote over empiricism”* ( Scott, 2005). Despite this skepticism, treatment resistance is one of the commonest indications for ECT in schizophrenia across the world ( Pompili *et al.*, 2013; Phutane *et al.*, 2011). Thus, ECT is commonly prescribed worldwide for an indication with inadequate evidence.

We planned this randomized sham-controlled trial to evaluate the efficacy of ECT in patients with schizophrenia resistant to clozapine or patients who did not tolerate clozapine (hereafter termed as clozapine-intolerant schizophrenia) and identify the predictors of response to assist in individualizing treatment. Hence, we plan to conduct a concurrent assessment of neuroplasticity measures along with multilevel neurobiological parameters and clinical symptom scores, which would enable disease modelling; furthermore, combining these measures with carefully controlled non-invasive brain stimulation (NIBS) experiments might unravel mechanistic basis and prognostic biomarkers of ECT in this population.

### Objectives and hypotheses

Primary objectives:

1. To compare the efficacy of bifrontal modified brief-pulse ECT and sham ECT in clozapine-resistant/intolerant schizophrenia. We hypothesize that true-ECT would demonstrate a greater clinical response than sham-ECT.
2. To evaluate the resting state brain connectivity predictors of response to ECT in patients with clozapine-resistant/intolerant schizophrenia. We hypothesize that a differential pre-treatment resting brain functional connectivity signal will identify responders to ECT.

Secondary objectives:

1. To compare the neurobiological profile of clozapine-resistant/intolerant schizophrenia patients with matched healthy controls using multimodal data (brain imaging, electroencephalography [EEG], transcranial magnetic stimulation-transcranial direct current stimulation [TMS-tDCS] perturbation study metrics, heart rate variability [HRV] and neuroplastic gene polymorphisms).
2. To examine the differential effect of true-versus-sham ECT on the neurobiological profile of clozapine-resistant/intolerant schizophrenia patients using multi-modal data (brain imaging, EEG, TMS-tDCS perturbation study metrics, HRV) and potential interactions with neuroplasticity gene polymorphisms.
3. To evaluate the predictive utility of multi-modal data (brain imaging, EEG, TMS-tDCS perturbation study metrics, HRV and neuroplastic gene polymorphisms) in identifying clinical response to ECT in patients with clozapine-resistant/intolerant schizophrenia.

## Methods

### Trial design

We employ a parallel-arm double-blind randomized sham-controlled design with an allocation ratio of 1:1 to determine the differential efficacy of add-on electroconvulsive therapy to sham-electroconvulsive therapy in clozapine-resistant/intolerant schizophrenia.

### Study setting

This would be a hospital-based study. The study would be conducted in patients with clozapine-resistant/intolerant schizophrenia at three mental health care and research institutes in India: National Institute of Mental Health and Neurosciences (NIMHANS), Bengaluru; Central Institute of Psychiatry (CIP), Ranchi; and Kasturba Medical College (KMC), Manipal. These study centers have been providing clinical services of ECT for more than 3–6 decades. The study would be centrally coordinated at NIMHANS.

### Eligibility criteria

Clozapine-resistant/intolerant schizophrenia patients who have been recommended and referred for ECT by the treating clinicians and already consented for receiving ECT would be approached for willingness to participate in this research study. Diagnosis made by the clinicians would be confirmed by an independent clinician using the structured clinical interview for DSM-5 (Diagnostic and Statistical Manual of Mental Disorders, Fifth Edition) disorders, clinician version (SCID-CV) ( First *et al.*, 2016).

Right-handed (assessed using Edinburgh Handedness Inventory ( Oldfield, 1971) patients with treatment-resistant schizophrenia who have shown resistance or intolerance to clozapine and are currently maintained on a stable dose of antipsychotic for a minimum six weeks but having persistent psychotic symptoms would be included in the study. Only consenting patients would be recruited after ascertaining the capacity to consent for participation in this research study using the University of California, San Diego Brief Assessment of Capacity to Consent (UBACC) ( Jeste *et al.*, 2007). This involves the capacity to understand the implications of taking part in the trial, including the sham-ECT part. Family members would be involved in the consenting process, but the final decision about participating in the study would be made by the participant themselves.

Treatment-resistant schizophrenia would be defined by inadequate response to at least two trials of antipsychotic (other than clozapine). The antipsychotics should have been provided at a minimum dose equivalent to 600 mg/day of chlorpromazine and/or 15mg/day equivalent of olanzapine calculated based on the classical mean dose method ( Leucht *et al.*, 2015). Each of these trials should have been for a minimum of six weeks with a minimum 80% adherence to prescribed antipsychotics (ascertained using at least two of the methods: patient report, caregivers report, case records, dispensing documents and serum levels wherever available). The first antipsychotic trial may be considered a failure with only a three weeks trial if an absolute lack of improvement is noted ( Taylor *et al.*, 2021). Inadequate response to these antipsychotic trials would be assessed based on a score of ≥ 3 in retrospectively assessed clinical global impression-improvement (CGI-I) or, wherever available, less than 20% improvement by the antipsychotic trial in a prospectively rated positive symptom score using a standard rating scale. If greater than “minimal improvement” on CGI-I or more than 20% improvement was observed, then non-response would be stated only if moderate or higher severity of positive psychotic symptoms persisted even after 10–12 weeks of the trial.

Such treatment resistant schizophrenia patients who are prescribed clozapine but were found to be having persistent psychotic symptoms would be included if they are found resistant or intolerant to clozapine. A patient on adequate doses of clozapine (serum clozapine level of >350ng/ml or minimum clozapine dose of 250 mg/day) for minimum three months would be considered clozapine resistant if they continue to have moderate or more severity of psychotic symptoms ( Howes *et al.*, 2017; Suhas *et al.*, 2020). The psychotic symptom severity eligibility will be defined by a score of ≥4 on one or more of the following items of brief psychiatric rating scale (BPRS) - Item 4 (Conceptual disorganization), Item 11 (suspiciousness), Item 12 (hallucinatory behaviour) or Item 15 (unusual thought content) ( Overall & Gorham, 1962) OR a score of ≥12 on total of the above items OR presence of at least two items in Bush Francis Catatonia Rating Scale screener ( Bush *et al.*, 1996). Those who discontinued clozapine due to poor tolerability or in whom the above-mentioned dose could not be administered would be considered clozapine intolerant schizophrenia. The intolerance/inability to increase the clozapine dose further will be based on a shared decision taken by a psychiatrist and user/care-giver. Stable antipsychotic dosage would be defined as less than 25% of change in the antipsychotic dose in six weeks preceding initiation of intervention.

Table 1 shows the full eligibility criteria for participants.

**Table 1.** Participant eligibility criteria.

Inclusion criteria 1. Schizophrenia Diagnosis (DSM-5) 2. Age 18 – 60 years 3. Any sex 4. Right-handedness 5. Treatment resistant schizophrenia 6. Resistant/intolerant to clozapine 7. On stable antipsychotic dosage 8. Moderate or more severity of psychotic symptoms 9. Prior consent by the patient for receiving ECT as treatment (obtained by the treating clinical team) ( Arumugham *et al.*, 2022) 10. Intact capacity to consent for research studies 11. Written informed consent ( Arumugham *et al.*, 2022)	Exclusion criteria 1. Severe general medical/neurological comorbidity that precludes ECT or has an effect on cognition and behaviour 2. Score of > 6 on the Calgary Depression Rating Scale ( Addington *et al.*, 2014) 3. Suicidal risk (HAM-D suicide item score>2)/any psychiatric emergency 4. Pregnancy / Post-Partum 5. Current psychoactive substance dependence (except caffeine or nicotine) 6. Co-morbid neurological/medical disease that can affect the brain structure/function 7. Any contraindication for magnetic resonance imaging 8. Any contraindication for transcranial magnetic stimulation 9. Any contraindication for transcranial direct current stimulation

### Intervention

**True ECT**: Thrice-weekly, bifrontal ECT would be provided for a minimum of nine sessions and would be extended till a plateau is achieved in clinical response. ECT would be provided with a NIVIQURE ECT device (current: 800mA, pulse frequency: 75 pulses per second, pulse-width: 1ms). Modification would be provided with short general anaesthesia and muscle relaxants (usually with thiopentone 2–4 mg/kg & succinylcholine 0.5 mg/kg). Alternative modification agents would be considered if there are any medical indications. The electrodes would be placed 5 cm above the outer canthus of each eye, along a vertical line perpendicular to an imaginary line connecting the two pupils. ECT would be administered as per the standard procedure by a team of psychiatrist(s), anaesthesiologist, nursing and other paramedical professionals.

The seizure threshold would be determined during the first ECT session through titration method. The stimulation would be initiated at 30mC with increments of 30mC until adequate seizure is achieved. The ECT administrator would initiate the stimulation at a higher dose depending on age and co-administration of anticonvulsants. From the subsequent sessions, patients would receive 1.5 times suprathreshold ECT. EEG would be recorded during the ECT with the NIVIQURE ECT–EEG device from left and right frontal pole leads (Fp1 and Fp2), referenced to bilateral mastoid processes. As per current guidelines, seizures would be considered adequate if there is evidence for generalized seizures observed in either the motor system (in the isolated limb) or in the typical morphology of EEG ( Ferrier & Waite, 2019). ECTs may be continued after nine sessions, if there are persisting symptoms and continuous improvement is observed.

After the 9th ECT session, ECT course would be terminated on achieving plateau in clinical response, i.e., if there is no change in the clinical status from the previous session in three consecutive sessions as measured by Sheehan-Clinician Global Improvement Global-21 scale (S-CGI-21) ( Sheehan *et al.*, 1993). Irrespective of the number of ECTs received, ECT would be terminated in any of the following situations: (1) Complete remission of symptoms; (2) significant cognitive impairment (as decided clinically) despite reducing the frequency to twice weekly; (3) medical conditions precluding continuation of ECT; (4) on request from the patient. The patients will be free to withdraw from the study at any point in time – the reason for withdrawal would be documented; (5) if patients develop suicidality amounting to HAM-D suicide item >2. A shared decision for discontinuation would be made after discussing with patients and the treating psychiatrist. 

**Sham-ECT**: Anaesthesia and muscle relaxation would be provided as discussed above, without electrical stimulus. ECT administrator would run the ECT stimulator by keeping electrodes in air, 1–4 times on the first ECT and 1–4 times in subsequent ECTs at a gap of 20–40 secs by setting the incremental electrical parameters. Patients in the sham ECT group would be offered an open-labelled true ECT, if symptoms persist at the end of the blinded trial. Open-labelled true ECT would be offered to patients who withdraw consent from the sham ECT arm due to lack of response.

***Adherence to intervention.*** All sessions would be provided at the hospital by the ECT administration team. The project would cover the hospital expenses of patients. They would also be reasonably compensated for their time and other expenses. Thus, the financial burden of participation in the trial would be minimal. Adverse effects would be monitored diligently, and suitable action would be taken to prevent discomfort. The research team would be available for any clarification/concern regarding the study through easily accessible means (like mobile phone, email). All these measures would encourage the adherence to the intervention and study retention.

***Concomitant care.*** Violation with study guidelines like medication change due to unforeseen situations would be noted down and reported. A change in antipsychotic dose by more than 25% from the dose at the initiation of the intervention, change in antipsychotic medication or initiation of other neuromodulatory interventions would be considered as drop-out. All on-going antipsychotic medication and psychosocial interventions concomitant to the study intervention would be allowed.

### Outcomes

Primary outcome would be measured as the change in scores from baseline to the end of 9 ^th^ session or to the point of premature termination of trial. A comparison of change in Scale for the Assessment of Positive Symptom Scale (SAPS) between the two groups would be the primary outcome measure. Response would be defined as ≥ 40% improvement in SAPS scores ( Petrides *et al.*, 2015), which is similar to the criteria employed in previous ECT studies in schizophrenia ( Ithal *et al.*, 2021).

Secondary outcome measures include other relevant clinical symptoms, adverse effects and neurobiological parameters (the detailed protocol of neurobiological parameters will be described in a separate paper). Time to response would be compared between the two groups. Clinical symptoms would be measured by SAPS, Scale for the Assessment of Negative Symptoms (SANS) ( Andreasen, 1982), BPRS ( Overall & Gorham, 1962) and Auditory Hallucination Rating Scale (AHRS) ( Hoffman *et al.*, 2005) at baseline and every week till the end of the intervention. S-CGI-21 after each ECT (true or sham ECT) session. Functional abilities will be assessed using Groningen Social Disability Schedule (GSDS)( Wiersma *et al.*, 1988) and social and occupational functioning scale (SOFAS) ( Morosini *et al.*, 2000) at baseline and after each step of intervention. All clinical symptom assessments after baseline will be done 24 hours after the respective ECT session.

Side-effects would be monitored using an ECT-side effect checklist after every ECT. Cognition would be monitored using a weekly Battery for ECT-Related Cognitive Deficits (B4ECT-ReCoDe) ( Viswanath *et al.*, 2013) and Montreal Cognitive assessment (MoCA) from baseline till the end of the trial. Brief Assessment of Cognition in Schizophrenia (BACS) ( Keefe *et al.*, 2004) would be done at baseline and end of the trial. Neurobiological assessments would be performed at baseline and after the end of RCT.

### Participant timeline ( Figure 1)

***Patients***

**Step-1:** Patients prescribed ECT by the treating psychiatrist fulfilling eligibility criteria will be approached for participation in the trial. Consenting participants would be randomized to true/sham ECT administered thrice-weekly for nine or more sessions till achievement of plateau in clinical improvement. Pharmacotherapy will remain stable during the ECT course.

**Figure 1.**
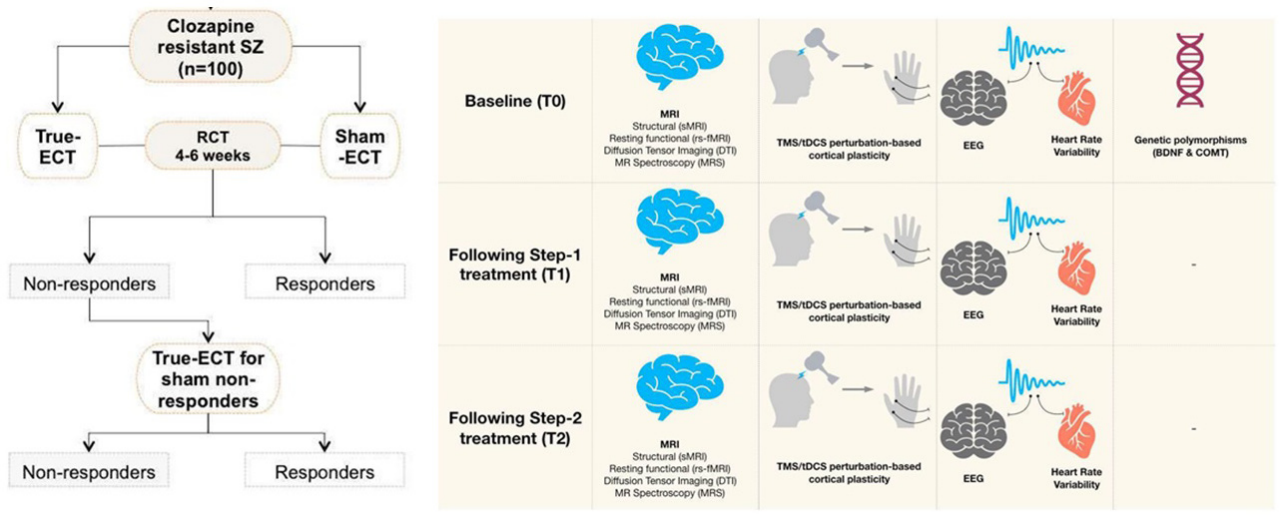
Depiction of study procedure. In the first step of the study, the patients with clozapine resistant or intolerant schizophrenia prescribed electroconvulsive therapy by the clinicians would be randomized to receive 4–6 weeks of True-ECT or Sham-ECT. Patients without clinical response by the end of the intervention in the sham-ECT group would be offered open-label true-ECT in the second step of the study. Neuroimaging, neurophysiological tests would be done at the baseline and end of each step. Blood samples would be collected for genetic polymorphism assays at the baseline. SZ: Schizophrenia, ECT: Electroconvulsive therapy, MRI: Magnetic resonance imaging, EEG: Electroencephalogram, TMS/tDCS: transcranial magnetic stimulation/transcranial direct current stimulation, BDNF: Brain derived neurotrophic factor, COMT: catechol-O-methyltransferase.

**Step-2:** Non-responders (<40% change in SAPS scores in step-1) of sham ECT group or patients who opted for premature termination of trial due to lack of response to the intervention in the step-1 will be given the option to receive true ECT, at the same frequency and similar duration as described in the step-1 of the trial. Written informed consent will be obtained separately prior to the initiation of both steps ( Arumugham *et al.*, 2022). All the clinical and neurobiological assessments would be repeated in the same frequency as in the step-1 of the study. 

### Sample size

A total of 100 patients would be recruited for the study. A meta-analysis has found an effect size of 0.88 for clozapine with ECT as compared to clozapine alone ( Wang *et al.*, 2018). The sample size for this study was calculated using a more conservative estimate of effect size 0.6 for a sham-controlled trial, to account for potential placebo response in the sham group. Given an effect size of 0.6 between the treatment groups for change in positive symptom severity, a minimum sample of 45 in each group would have 80% power to detect post-treatment differences between the two groups with an alpha of 0.05 in a two independent sample t-test. In order to account for dropouts, we propose to recruit 50 patients in each group. For comparison studies between patients and healthy controls, a sample size of at least 100 healthy subjects would offer optimal power as per the sample size of previous neurobiological studies in this area ( Goyal *et al.*, 2011; Mouchlianitis *et al.*, 2016; Raymond *et al.*, 2022).

### Allocation

***Sequence generation.*** Consenting patients would be randomly assigned using computer-generated codes to true/sham ECT. Random allocation of two treatments (ECT and SHAM) for three study centers with unequal strata sizes of 60, 20 and 20 would be carried out in Stata ver.15.1 ( *StataCorp.,* 2017). This procedure would provide a sequence of treatments (ECT & SHAM), which would be randomly permuted in blocks of varying sizes and order. An independent statistician would manage the randomisation and allocation concealment. The allocation sequence generation will be centrally coordinated at NIMHANS.

***Concealment mechanism.*** Allocation concealment would be ensured through sequentially numbered sealed opaque envelopes. The person administering ECT would allocate the study arm by opening the sealed envelope on the first day of intervention after anaesthesia and muscle relaxant are administered.

***Masking.*** The participants, their care-givers, investigators, safety assessors, outcome assessors, treating psychiatrists, data managers and data analysts would be blinded to the allocated study arm. Staff involved in the ECT administration including an independent psychiatrist (not involved in regular clinical care of the patient), anaesthesiologist, and paramedical staff would become unblinded thereafter. Paramedical staff in the recovery room would remain blinded to the study arm and would assess the time to reorientation and post ECT adverse effects. Independent assessors would monitor study outcome and they would remain blinded about the study arm till the end of post-RCT assessments. As the risk of unblinding would be high on immediate post-ECT adverse effect evaluation, outcome assessors would remain blinded to the immediate adverse effects. The treating clinical team would also remain blinded to study arm and immediate cognitive/behavioral adverse events till the end of post-RCT assessment. To maintain the rigorous blinding, two independent assessors would rate the safety and outcome measures.

ECT related records will be maintained by the ECT administrators who are unblinded to the study. ECT procedure details including the details of seizures will be maintained in the ECT suite securely by the ECT administrators. The device used in this study shows an online digital display and provides a digital copy of the EEG which will again be securely stored by ECT administrator. Adverse outcome assessors maintain the adverse effect assessment records securely under the supervision of the psychiatrist administering ECT. This psychiatrist will decide on intimating the adverse events to the clinical teams without compromising the clinical needs of the patients and maintaining the blinding as much possible.

***Emergency unblinding.*** Serious adverse events (SAE) would be reported to the trial management group (TMG) and data and safety monitoring board (DSMB), which would assess for suspected unexpected serious adverse reaction (SUSAR); the patient, caregiver, clinical team and the investigators may be unblinded at their discretion. Otherwise, all participants would be unblinded at the termination of the trial (either after completion of nine sessions or premature termination for any other reason). Assessment of blinding efficacy would be done on patients, adverse event assessors and outcome assessors after the last (scheduled/terminated) blinded intervention session using a 5-point likert scale ( Bang *et al.*, 2004).

***Trial procedures and evaluations.*** In addition to the above discussed screening tools and outcome tools, all participants would be assessed using a comprehensive semi-structured proforma to collect the socio-demographic and clinical details would be collected ( Arumugham *et al.*, 2022). Clinical ratings would be performed by mental health professionals. They would be trained in administering the clinical rating scales and inter-rater reliability assessments would be performed for the primary outcome measure. Comprehensive data collection forms would be available with the investigators and would be made available on reasonable request.

**Figure 2.**
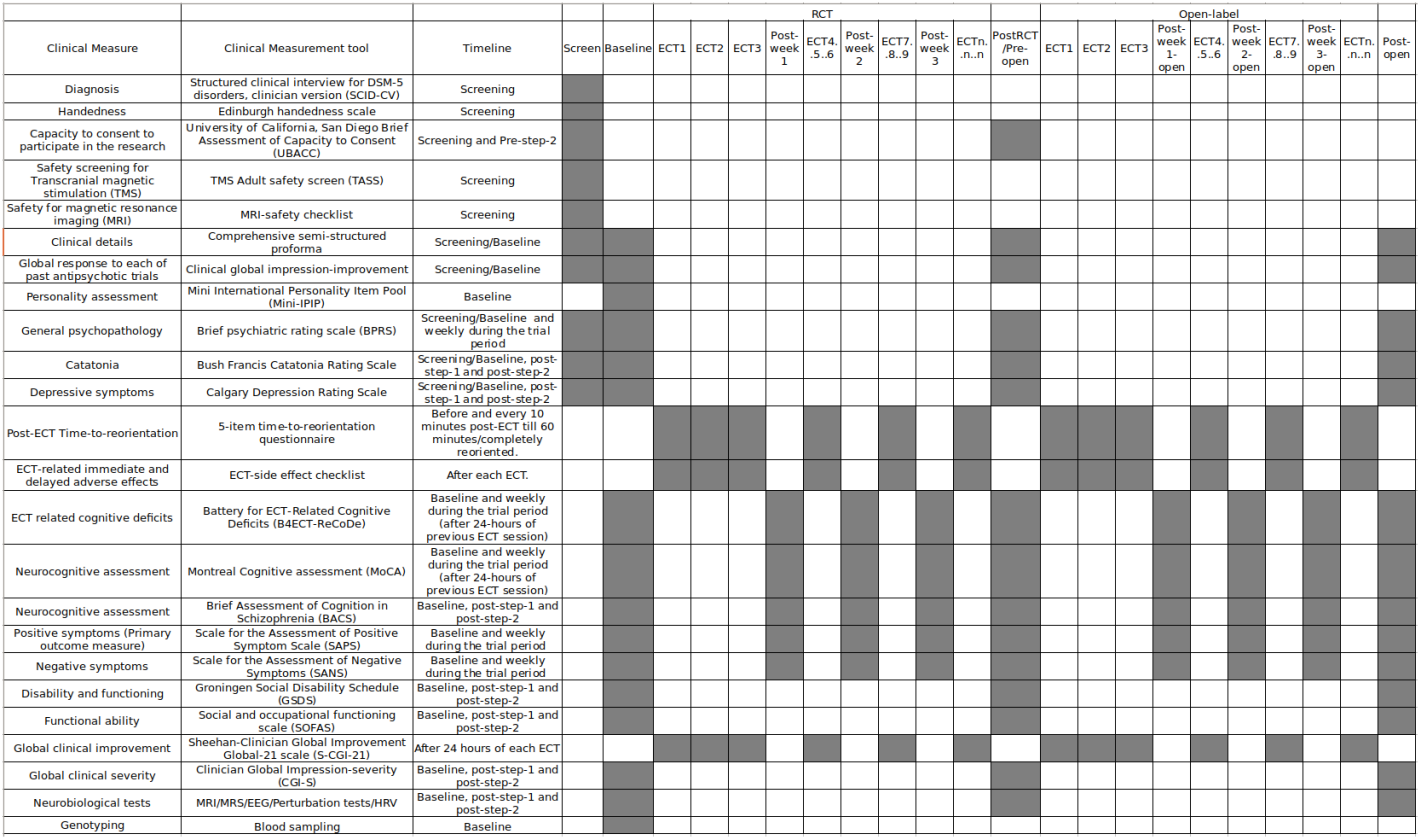
Gantt chart of the assessment timeline in patients. Grey boxes are scheduled timepoints of respective assessments. ECT _n_= n ^th^ ECT session. *Weekly assessments during the course of treatment will be done every week even if frequency of ECT is reduced to 2/week.

Multi-modal neurophysiological data (magnetic resonance imaging [MRI], EEG, cortical perturbation using TMS-tDCS, HRV) would be acquired before and after interventions to identify predictive factors and understand mechanistic basis of the ECT outcome. Clinical and neurobiological evaluations would be repeated even after the end of the open label phase. Also, neuroplasticity gene polymorphisms (brain derived neurotrophic factor [BDNF] and catechol-O-methyltransferase [COMT] genes) will be assessed alongside clinical and neurophysiological data to identify the predictors of clinical response. At least 100 subjects matched (as a group) for age, sex, education, handedness, and socio-economic status would be chosen for the comparative analyses of neurobiological studies. They would undergo a one-time baseline assessment of clinical and neurobiological parameters. Figure 2 depicts the assessment timepoints.

### Neurobiological investigations

**MRI studies:** MRI data will be acquired with a 3T scanner (Ingenia CX, Philips). Structural MRI, resting-state functional MRI and Diffusion Tensor Imaging will be acquired as per the established protocol ( Parekh *et al.*, 2021). Magnetic resonance spectroscopy studies will be done to measure glutamate levels in the left dorsolateral prefrontal cortex and left temporoparietal junction based on their pathophysiological role in schizophrenia ( Fan *et al.*, 2017; Younis *et al.*, 2020).

**Cortical reactivity and plasticity:** Motor cortical reactivity will be determined by employing single and paired-pulse TMS-EMG investigational approaches to determine the motor threshold, silent period, and intracortical inhibition/facilitation. We will also employ perturbation-based motor cortical plasticity experiments to quantify the magnitude and direction of change in motor evoked potentials with a single session of iTBS/cTBS/c-tDCS. The type of perturbation protocols used will mimic those used in the actual treatment protocols ( Mehta *et al.*, 2019).

**EEG:** Continuous resting-state EEG will be acquired for 20 minutes. Sixteen minutes of eyes-closed resting-state EEG would be acquired in two separate time blocks of 12 minutes and 4 minutes with an intervening 4-minute duration of an eyes-open EEG. EEG data will be acquired by a 64-channel amplifier with the electrode montage placed based on the international 10/10 system. All data will be referenced against an electrode centred on the midline between Fz and Cz, and sampled at 5 kHz ( Babiloni *et al.*, 2020; Trujillo *et al.*, 2017)

**HRV:** 15-min resting ECG will be obtained to determine the beat-to-beat variability of heart rate, and the multitude of both time (SDNN, RMSSD, PNN50, triangular index) and frequency domain (low and high frequency) measures of HRV ( Task Force of the European Society of Cardiology and the North American Society of Pacing and Electrophysiology, 1996).

***Blood assay.*** Blood samples will be collected from the ante-cubital vein into K _2_ EDTA vacutainer tubes (Becton & Dickinson, U.S.A). DNA extraction will be carried out using the commercial spin column method (Qiagen, Inc.). Extracted DNA will be checked for quality assurance and stored at -80°C for later use. PCR amplification for SNP assays of selected functional polymorphic variants of COMT ( Chhabra *et al.*, 2018) and BDNF ( Strube *et al.*, 2014) genes will be performed. 

The details of the neurobiological data acquisition protocol will be described in a separate paper. 

### Data management

The multi-level data acquired from the enrolled participants would be quality checked and anonymised so that subject identifiers are removed. These data would be stored with a coded identification. Different streams of data (ex. Clinical, MRI, EEG etc.,) would be linked using codes to facilitate seamless retrieval of different data types.

### Data analysis

Anonymized coded data would be used for analysis. The primary outcome would be assessed as the change in SAPS scores from baseline to post-9th session of treatment using a two-sample t-test. The last observation carried forward method would be used to handle the missing data of those participants who had premature termination of the intervention. The percentage of responders between the two groups would be compared using logistic regression with estimates of adjusted odds-ratio (adjusting for variables including age, sex, duration of illness, antipsychotic daily dose, and anticonvulsant medications). Further, comparison of change in the symptoms scores between the two groups at the end of the interventions would be performed through linear mixed-effects model analysis. The time to response would be compared between the two groups using survival curves. Suitable imputation methods would be used for any missing data at specified time-points. Secondary outcome measures including other psychopathology rating scales, adverse effects and dropout rates would also be compared between the two groups.

The pre-processing of neurobiological data (where applicable – MRI/EEG data) will be done according to the internationally accepted processing pipelines. Data analysis will be carried out in two phases – a) association and b) prediction. The first phase will involve exploratory analyses with biographic (age, sex, etc.) and disorder-specific (including age-at-onset, medication, the severity of symptoms) features that influence MRI, EEG, TMS-derived neuroplasticity metrics, HRV, and gene polymorphism measures. These include traditional biostatistical procedures such as descriptive statistics and hypothesis testing for associations in order to identify any potential biomarkers of clinical response. The second phase of analysis will involve building predictive models that will be validated with an independent dataset. Here, instead of examining which individual measurements correlate with outcome measures, we seek to identify combination features (from resting-state fMRI as the primary objective; combining across data modalities will be the secondary objective) that can give the best prediction for the clinical outcome of proposed brain stimulation methods. State-of-the-art feature engineering/machine learning methods, such as gradient boosting/deep neural nets, will be used to train the model on a part of the acquired dataset. Lastly, causal models like Bayesian networks or mediational analyses will be explored to uncover potential causal relationships between study variables.

### Data monitoring

***Formal committees.*** The DSMB, an independent advisory body, would evaluate the data during the course of the study contributing to the scientific and ethical integrity of the study. It would periodically review and evaluate clinical efficacy and safety data collected during the study, and assess reports on cumulated SAE. It consists of a biostatistician, psychiatrist, and ethicist with a specialization in psychiatry. The board, based on the DSMB charter, will provide a report of recommendation on the continuation, modification, suspension, or termination of the trial to the study sponsor. The sponsor will submit the report to the ethics committees and the TMG to decide the next course of action.

The TMG will comprise a group of investigators from the three collaborating institutes. It will monitor the day-to-day execution of the trial. It would take cognizance of trial participant drop-out in the event of withdrawal of consent, noncompliance with study guidelines, and worsening of symptoms. It would interact with the DSMB and Institute Ethics Committee (IEC). It would review the reports and recommendations of these oversight/monitoring committees and plan appropriate actions. It would decide on trial terminations and any trial modifications of the study procedures. It would also oversee the submission of modification, premature termination (if any), and final (completed) trial summary report to the IEC, trial registries, and funding agency.

The data management committee comprises the principal investigators of the three institutes. It would be dedicated to reviewing the data archival, management, and sharing. It would execute the periodic data auditing and audit of the research practices related to the studies. It would ensure anonymization and de-identification of data. It would monitor the storage of data in the encrypted data storage servers. It would oversee the sharing of de-identified data upon reasonable requests from collaborators or the research community. Public access would be granted as per the policy of DBT-Wellcome trust India Alliance and international good research practices in strict compliance with the ethics guidelines for biomedical research in India.

### Interim analysis

We plan to conduct an interim analysis of the data after 20 patients in each group have been randomized. This will be shared with the DSMB and if it observes that there is a clear disadvantage to the sham-ECT patients then this would be brought to the notice of the ethics committee. A decision to continue the trial would be revised to avoid exposure of more patients to a less effective treatment by TMG. If a clear level of adversity from ECT proves to be above a predefined threshold that is clinically significant as deemed by DSMB and TMG, the trial would be discontinued to prevent any undue harm to patients in consultation with the ethics committee.

### Safety and harm

All participants would be assessed daily for clinical changes. S-CGI-21 would be applied a day after each ECT session. Patients will be closely monitored for any adverse effects using checklists and tests as described previously. SAE would be reported to the TMG and DSMB, which would assess for SUSAR. In an unlikely event of a harm attributable to participation in research procedures, financial compensation would be provided as per the provisions of the National Ethical Guidelines for Biomedical and Health Research involving Human Participants by Indian Council of Medical Research 2017 ( Mathur & Swaminathan, 2018).

### Ancillary care and post-trial care

In the event of withdrawal of consent, non-compliance with study guidelines, and worsening of symptoms, the concerned participant will be dropped from the trial. The best practiced standard care at the respective institutes would be provided by the treating teams of the patients even after the trial.

### Auditing

Periodic auditing of data and the research practices will be conducted by trial management group and data management committee. They would ensure the quality of research and patient advocacy.

### Ethics

***Ethics approval.*** Requisite clearances have been obtained from the IECs of all three institutes [NIMHANS: Ni.NIMHANS/EC (BEH.SC.DIV.)23 ^RD^ MEETING/2019-20 dated 07/04/2020; CIP: No.IEC/CIP/2020-21/337 dated 22/05/2021; KMC: IEC:109/2020 dated 12/02/2020], as well as from an ethics committee not affiliated to any of the three institutes (MS Ramaiah Medical College Ethics committee; MSRMC/EC/AP-09/06-2020). The trial is registered in Clinical Trial Registry of India (CTRI; Reg no: CTRI/2021/05/033775 registered on 24 ^th^ May 2021).

***Protocol modification.*** Upon interim analysis or during the course of the study, any modification made to the study protocol will be reflected in the trial registry (CTRI), and will be intimated to the Institute Ethics Committee, India Alliance CPH Committee (Funding Agency) and *Wellcome Open Research* (where the study protocol has been published).

***Informed consent.*** Trained health professionals will obtain consent from the participants ( Arumugham *et al.*, 2022). Participants would be assessed for their capacity to consent for the study, based on capacity to consent for research studies as per the assessment using the University of California, San Diego Brief Assessment of Capacity to Consent (UBACC). Only such patients who can understand the implications of taking part in the trial, including the sham-ECT part, would be recruited. Family members would be involved in the consenting process, but the final decision about participating in the study would be made by the participant themselves.

Additional consent for using participant data for advanced analysis, future research studies and sharing of coded and de-identified data with interested national and international researchers/collaborators following the regulatory guidelines would be taken ( Arumugham *et al.*, 2022).

### Access to data

The centralized data repository would be created and maintained at NIMHANS that would archive de-identified and coded data from all three institutes. The data management committee of investigators of three institutes will review data archival, management and sharing.

### Dissemination policy

A clinical trial summary report will be prepared and provided to the Institute Ethics committee and trial registries within 12 months from the completion of the study. A final (completed) trial summary report will be submitted to the funding agency for perusal. De-identified data would be shared with the research community and public access would be granted as per the policy of DBT- Wellcome trust India Alliance ( “India Alliance - Advancing Discovery & Innovation to Improve Health,” n.d.) and international good research practices in strict compliance to the ethics guidelines for biomedical research in India ( Mathur & Swaminathan, 2018).

Encrypted data storage servers would host the multi-modal clinical and neurobiological, including genetic data of all participants. The findings of the research would be disseminated primarily through publication in peer-reviewed journals. Prior to the publication, research data may be presented at national and international scientific fora. The study result and interpretation as a report would be shared with all major stakeholders. The team will engage in regular interaction with all stakeholders (psychiatrists in community, patients and caregivers, policy makers, regulatory bodies, other psychiatry institutes and industry) for dissemination of knowledge gathered from the trial.

Publications from the trial will acknowledge that the work has been carried out by all participating institutes (NIMHANS, CIP & KMC). The authorship will be in strict adherence to the ethical and research publication guidelines ( Resnik *et al.*, 2016). There is no intention of using professional writers in any of these works.

## Sponsor information

The DBT-Wellcome Trust India Alliance is the funding agency. It has constituted a committee of technical experts who would periodically review the trial progress, research practices, outputs and accountable utilization of funds. The Clinical research center for neuromodulation in Psychiatry consists of the three participating institutes (NIMHANS, Bengaluru; CIP, Ranchi; and KMC, Manipal). The Heads of the institutes of the three institutes are the sponsors. The funders and sponsors would support the project through expert suggestions through the technical committees but would not be involved in study design; collection, management, analysis, and interpretation of data; writing of the report; and the decision to submit the report for publication. The investigators hold the final authority on taking these decisions. 

## Discussion

Schizophrenia accounts for a large proportion of patients receiving ECT, which is up to 85% of all cases posted for ECT in Russia ( Leiknes *et al.*, 2012). Schizophrenia is the most common indication for ECT in the most populous regions of the world i.e. Asia, Africa and South America ( Leiknes *et al.*, 2012), yet there is nearly eight-fold difference in the quantum of ECT research on schizophrenia vis-à-vis that in depression ( Mahadevaiah *et al.*, 2010). ECT is a major procedure that involves repeated exposure to anaesthesia and electrically-induced seizures and associated risks and adverse effects, including unpleasant cognitive deficits. ECT is also fairly expensive and often requires inpatient care. Patients and relatives have to spend several half days for ECT sessions on outpatient based ECTs. It is clear that thousands of patients with schizophrenia receive ECT without the presence of robust evidence to support this practice. It is difficult to refute the possibility that these patients may be receiving ineffective treatment with substantial risks and adverse effects. In the absence of a well-designed randomized double-blind sham-controlled trial of ECT in clozapine-resistant schizophrenia, they would continue to receive ECT without a firm evidence base to support this practice or they would be left without any other evidence-based treatment.

It is not clear whether patients who experience improvement with ECT do so because of several factors associated with the procedure, placebo response, natural course of illness or continuation of their medications. Therefore, only a sham-controlled trial could conclusively demonstrate the efficacy of ECT. A Cochrane review in 2019 identified 14 of the 15 studies on ECT in treatment-resistant schizophrenia as having a high risk of bias, primarily owing to blinding related issues. The only sham-controlled study included in the review did not show significantly difference in improvement in the symptom severity between the sham and true ECT arms ( Sinclair *et al.*, 2019). The challenge in conducting a controlled-trial with ECT is with respect to the sham-ECT component. Although administration of sham ECT is challenging due to the risk of anaesthesia and muscle relaxants, it is needed for adequate blinding of participants and control for placebo effects. About half of the participants in the trial would receive sham-ECT. A proportion of the participants in the sham-ECT arm may improve without electrically-induced seizures; they are likely to experience fewer adverse effects than those in the true-ECT arm. The risks of anaesthesia are nearly the same for participants in either limb. Only such patients, for whom their treating clinicians believe that ECT is indicated, would be recruited in the study. In other words, subjects who are likely to take part in this study would have received ECT even if they were not part of this study. No patient in whom ECT is not a reasonable option would be recruited. Also, participants would be assessed for their capacity to consent to the study. Only such patients who can understand the implications of taking part in the trial, including the sham-ECT part, would be recruited. Family members would also be involved in the consenting process, but the final decision about participating in the study would be made by the participant him/herself.

Those who receive sham-ECT and do not experience improvement would be offered a course of true ECT as part of the study. Patients with schizophrenia often receive long courses of ECT; particularly, those with treatment-resistant schizophrenia receive around 20 sessions of ECT ( Zervas *et al.*, 2012). Therefore, the administration of nine sham sessions followed by an open-label treatment up to nine sessions as part of the current trial would be well within the current practice of ECT in resistant schizophrenia. In addition, we plan to conduct an interim analysis of the data after 20 participants have been randomized. If we observe that there is a clear disadvantage for sham-ECT participants, then this would be brought to the notice of the IECs and DSMB, and a decision to continue the trial would be revised to avoid exposure of more participants to a less effective treatment. Participants have the right to withdraw from the trial at any point in time. Thus, those who are not satisfied with the level of improvement are free to drop out of the study. We hope that this would address the concerns regarding participants in the sham-ECT limb experiencing less efficacious treatment.

One of the major limitations of the study would be exclusion of severely ill patients who may be having high suicidality or other psychiatric emergency conditions where ECT is known to benefit and those who would lack the capacity to consent to participate in the research. This could be a major subset of patients who receive ECT in clinical settings and this limits the generalisability of the study. The study would be provided as an add-on to the ongoing pharmacotherapy and psychosocial interventions. However, these treatment schedules and doses would be kept stable and would replicate the general clinical care setting where ECT is provided for this group of patients.

We hope that the result of this trial will be able to provide a definitive answer on the efficacy of ECT in clozapine-resistant schizophrenia. This study would provide clues to predictive neurobiological and clinical markers. Therefore, it would enable clinicians to take evidence-based decisions in managing such patients.

## Data Availability

No data is associated with this article. Open Science Framework: Consent form, ECT Information booklets and semi-structured proforma for ‘Clinical efficacy and neurobiological correlates of electroconvulsive therapy in patients with clozapine-resistant/intolerant schizophrenia: study protocol of multi-site parallel arm double-blind randomized sham-controlled study’. https://doi.org/10.17605/OSF.IO/J7QYM ( Arumugham *et al.*, 2022) This project contains the following extended data: Supplementary file 1: 01-Consent-Project-01-Revised.pdf Supplementary file 2: ECT consent Form with Versions.pdf Supplementary file 3: ECT information Brochure.pdf Supplementary file 4: Semi Structured Proforma_Study1_ECT in CRS.pdf Open science framework: SPIRIT checklist for ‘Clinical efficacy and neurobiological correlates of electroconvulsive therapy in patients with clozapine-resistant/intolerant schizophrenia: study protocol of multi-site parallel arm double-blind randomized sham-controlled study’. https://doi.org/10.17605/OSF.IO/J7QYM ( Arumugham *et al.*, 2022)
