# Supplementary material for: Clinical efficacy and neurobiological correlates of electroconvulsive therapy in patients with clozapine-resistant/intolerant schizophrenia: study protocol of multi-site parallel arm double-blind randomized sham-controlled study
Source: Wellcome Open Res. 2022 Oct 5;7:212. Originally published 2022 Aug 12. [Version 2] doi: 10.12688/wellcomeopenres.18028.2 (PMC10636350; doi:10.12688/wellcomeopenres.18028.2)
Supplement: Peer review history

# Reviewer report: Reviewer response for version 2

**DOI:** 10.21956/wellcomeopenres.20418.r68189

**Published:** 2023-11-09

**Authors:** Scott R Clark

**Affiliations:** 1 The University of Adelaide, Adelaide, South Australia, Australia

The authors have composed a detailed protocol that addresses a substantial gap in evidence regarding the efficacy for ECT for primary psychotic illness.

 The ethical issues and problems with blinding have been well described by other reviewers.

 The consent processes are robust but given levels of insight in this cohort, numbers consenting may be limited.

 Introduction:

 The introduction lacks a rationale for response biomarker studies.

 Methods:

 If the rationale for excluding left handed patients is related to imaging/EEG biomarker studies then could left hand patients be included in the main trial but excluded from biomarker studies?

 Is it feasible/ safe for the treating clinical to remain blinded to immediate cognitive/behavioral adverse events during ECT treatment? How will post ECT effects be fully blinded?

 Given the unique properties of clozapine will subsamples that are intolerant and not taking clozapine be analysed separately from those taking clozapine? What impact would this have on study power?

 Could the ECT protocol be more flexible to allow less frequent dosing (eg. 2 times a week) or shift to right unilateral placement if there is increased side effect burden but overall improvement?

 Will benzodiazepines and antiepileptic mood stabilisers be held during ECT?

 There is limited description of biomarker collection protocols and the burden on participants and feasibility of these protocols additional to that of the main study is not clear.

Is the study design appropriate for the research question?

Partly

Is the rationale for, and objectives of, the study clearly described?

Partly

Are sufficient details of the methods provided to allow replication by others?

Partly

Are the datasets clearly presented in a useable and accessible format?

Not applicable

Reviewer Expertise:

Treatment resistant schizophrenia, clozapine, electrophysiological biomarkers, TMS

I confirm that I have read this submission and believe that I have an appropriate level of expertise to confirm that it is of an acceptable scientific standard, however I have significant reservations, as outlined above.

# Reviewer report: Reviewer response for version 2

**DOI:** 10.21956/wellcomeopenres.20418.r52676

**Published:** 2022-10-12

**Authors:** Prashanth Mayur

**Affiliations:** 1 Royal Australian and New Zealand College of Psychiatrists, Melbourne, Australia; 2 Cumberland Hospital, Sydney, NSW, Australia

I have read the revised version of the manuscript. Most of the concerns and suggestions that I had raised have been addressed satisfactorily. I do not have any further comments and I would like to formally approve the article.

Is the study design appropriate for the research question?

Yes

Is the rationale for, and objectives of, the study clearly described?

Yes

Are sufficient details of the methods provided to allow replication by others?

Yes

Are the datasets clearly presented in a useable and accessible format?

Not applicable

Reviewer Expertise:

Electroconvulsive Therapy.

I confirm that I have read this submission and believe that I have an appropriate level of expertise to confirm that it is of an acceptable scientific standard.

# Reviewer report: Reviewer response for version 1

**DOI:** 10.21956/wellcomeopenres.19990.r52325

**Published:** 2022-09-09

**Authors:** Phern Chern Tor

**Affiliations:** 1 Department of Mood and Anxiety, Institute of Mental Health, Singapore, Singapore

This is an important and ambitious placebo controlled trial for the use of ECT augmentation in patients with highly treatment (clozapine) resistant schizophrenia.

 A previous reviewer has given an excellent review and I shall only add 3 additional thoughts.

 The evidence of a real effect of ECT in this population, while not as robust as in depression, is far from absent. The Phutane et al. and Bansod et al. papers show differential effect of varying ECT modalities in schizophrenia (BF, BT, RUL), suggesting a real effect of ECT rather than placebo. This should be addressed directly in the background and justification for the current study.

 The second and major concern I have is the blinding procedure as blinding of assessors will be key. The description of blinding / masking is quite brief and considering the complexity of the intervention over 3 sites, I am doubtful if full blinding can be maintained. A simple example of a problem would be the presence of absence of EEG tracings in the case file. It is likely that EEG tracings are printed out and attached to the patient file or electronic medical record. Other details like duration of motor EEG are often recorded in routine clinical practice. If these are missing, it is hard to truly blind the assessors. See the McManus paper for a detailed description of these challenges. Unless these are robustly addressed, the results of this study will be subject to challenge.

 The last concern is the key outcome of interest (SAPS) is not used for inclusion and rather a general screening questionnaire (BPRS) is used to assess symptom severity for trial inclusion. While there may be good reasons for this, it introduces complexity and scope for bias where patients score highly on the general screening questionnaire but perhaps not as high on the outcome questionnaire due to greater specificity of the outcome scales, or vice versa. The outcome scale should be used for the intake assessment as well to reduce any bias in the recruitment process.

Is the study design appropriate for the research question?

Partly

Is the rationale for, and objectives of, the study clearly described?

Yes

Are sufficient details of the methods provided to allow replication by others?

Yes

Are the datasets clearly presented in a useable and accessible format?

Yes

Reviewer Expertise:

Electroconvulsive Therapy

I confirm that I have read this submission and believe that I have an appropriate level of expertise to confirm that it is of an acceptable scientific standard, however I have significant reservations, as outlined above.

## References

[1] : Double-blind randomized controlled study showing symptomatic and cognitive superiority of bifrontal over bitemporal electrode placement during electroconvulsive therapy for schizophrenia. *Brain Stimul* .2013;6(2) : 10.1016/j.brs.2012.04.002 210-7 10.1016/j.brs.2012.04.002 22560048

[2] : A Randomized, Nonblind, Naturalistic Comparison of Efficacy and Cognitive Outcomes With Right Unilateral, Bifrontal, and Bitemporal Electroconvulsive Therapy in Schizophrenia. *J ECT* .34(1) : 10.1097/YCT.0000000000000454 26-30 10.1097/YCT.0000000000000454 28937546

[3] : Simulated Electroconvulsive Therapy: A Novel Approach to a Control Group in Clinical Trials. *J ECT* .2022;38(3) : 10.1097/YCT.0000000000000832 165-170 10.1097/YCT.0000000000000832 35220356PMC9420159

# Reviewer report: Reviewer response for version 1

**DOI:** 10.21956/wellcomeopenres.19990.r51970

**Published:** 2022-09-01

**Authors:** Prashanth Mayur

**Affiliations:** 1 Royal Australian and New Zealand College of Psychiatrists, Melbourne, Australia; 2 Cumberland Hospital, Sydney, NSW, Australia

This is a study that is ambitious in its scope of determining two specific matters 1) whether ECT is an augmenter of clozapine-resistant/intolerant schizophrenia at least in terms of positive symptoms and 2) whether neurobiological biomarkers, comprehensively assessed in this study, could predict ECT responders. The use of sham ECT is novel in the 21 ^st^ century and raises the ethical issue of delaying or temporarily denying an active treatment arm.

 Most of the specific comments below are minor except for a more nuanced discussion on the ethics of sham-ECT treatment in schizophrenia.

**Background and Rationale**

 The background and rationale are well written and highlight the limitations of high-quality research of ECT in combination with antipsychotics/clozapine in treatment-resistant/intolerant schizophrenia. The authors have made a compelling argument of the need for a sham treatment arm for ECT based on eliminating placebo effects. The reviewer suggests bringing in the ethical dilemma of sham-controlled arm at the outset, which would then be systematically addressed in the methods.

- The sentence, "Most people with this condition, the majority of whom are very young *…*" Suggest taking out "very young". This may not be the case in clozapine-resistant schizophrenia where there could be a greater age-spread with this condition.
- The sentence in paragraph 3 ("However, the improvement in the ECT group in this non-blinded study cannot be attributed to the direct therapeutic effect of ECT alone") *, *requires clarification as to why the authors came to this conclusion. In the same sentence, the Petrides et al 2015 study is not non-blinded but single-blinded. The raters were masked/single-blinded as mentioned earlier by the authors. The sentence should reflect this accurately.
- In the last paragraph, please expand the acronym NIBS.

**Objective, Hypothesis and Methods**

 The objectives are clearly stated. The primary objective on clinical positive symptoms efficacy with or without ECT is clear. The definitions of clozapine-resistant and intolerant schizophrenia patients are meticulous and exhaustive setting a high bar in research of this area. The methods are clear and meticulously stated with conservative power analysis for the primary outcome. There is a vigorous approach to blinding and sham-ECT delivery. The neurobiological assessments are comprehensive and cutting-edge in ECT research with schizophrenia. This is to be specially commended. It is highly likely that this study will significantly value add to the literature on brain mechanisms during ECT in schizophrenia.

 The use of the UBACC is an important addition to ensure an easy-to-use screening tool for decision-making capacity in research. Unfortunately, the study is limited in considering only patients who have a capacity to give consent thus eliminating an arguably larger number of more severely unwell patients. This is understandable and appropriate due to the sham-arm.

- Please clarify whether the second primary objective ("To evaluate the predictors of response to ECT in patients with clozapine-resistant/intolerant schizophrenia" *) *is different from the third secondary objective of determining predictive utility of a wide range of biomarkers to ECT response.
- Right-handed patients - The reviewer sees no clinical/ethical reason to not provide left-handed patients a similar chance to participate in the trial particularly when the ECT’s electrode placement is entirely bifrontal. The reviewer suggests omission of this criteria and inclusion of left-handed and ambidextrous patients in the study.
- Please clarify rationale for using a fixed stimulus frequency of 75pps to deliver the ECT stimulus. Why not 70pps or 80pps? This may be relevant in the study not allowing data generalisation with other ECT studies with devices such as MECTA and Thymatron where the stimulus frequency typically varies between 20Hz and 120Hz in 5-10 Hz intervals (or 10-20pps intervals).
- On Page 6, *“*The seizure threshold would be determined during the first ECT session through titration method *”. *Please clarify if there are additional criteria viz., EEG/Motor seizure length/quality to determine a threshold seizure. It would be appropriate to be specific.
- On Page 6, “After the 9th ECT session, ECT course would be terminated on achieving plateau in clinical response, i.e., if there is no change in the clinical status from the previous session in three consecutive sessions as measured by Sheehan-Clinician Global Improvement Global-21 scale (S-CGI-21) (Sheehan et al., 1993) *”.* Is this justified or necessary since the primary outcome is determined at the 9 ^th^ ECT session? Would this take away decision-making about termination on routine clinical grounds by the respective treating teams? Please clarify.
- Page 7, "Clinical symptoms would be measured by SAPS, Scale for the Assessment of Negative Symptoms (SANS) (Andreasen, 1982), BPRS (Overall & Gorham, 1962) at baseline and every week till the end of the intervention. S-CGI-21 would be assessed after 24 hours of each ECT (true or sham ECT) session." It is suggested that *all *the symptom assessments, after baseline assessments, are uniformly conducted at the same time point, 24 hours after each ECT, to avoid rater-bias of guessing the treatment arm related to the immediate cognitive effects of post-true ECT effects. The sentence should be modified to reflect this.
- Page 7, "Clinical symptoms would be measured by SAPS, Scale for the Assessment of Negative Symptoms (SANS) (Andreasen, 1982), BPRS (Overall & Gorham, 1962) at baseline and every week till the end of the intervention." The reviewer *s*uggests using an additional specific scale to measure auditory hallucinations (as a secondary outcome measure) specifically since ECT may have a differential impact on this measure. Treatment-resistant auditory hallucinations may be more susceptible to brain stimulation in comparison to treatment-resistant delusions or treatment-resistant formal thought disorder. This may be particularly important in this sham control trial where the sham treatment ends at 9 sessions to target specific sensitive parts of a heterogenous condition of schizophrenia. This is likely to provide valuable clues to future approaches in studies of ECT in schizophrenia. This will also add further novelty to this study.
- Page 7, "Functional abilities will be assessed using Groningen Social Disability Schedule (GSDS)(Wiersma et al., 1988) and social and occupational functioning scale (SOFAS) (Morosini et al., 2000) at baseline and after each step of intervention." The reviewer is not able to understand the relevance of both these scales, especially the SOFAS, while the study is entirely conducted in an inpatient population. It is expected that self-care and family interactions may improve during the intervention but to report on wider social and occupational functioning is stretching things too far. Suggest using just one of these two scales.
- On Page 11, under "Informed consent", while the UBACC is used to assess a patient’s decision-making capacity, did the participants and their families additionally receive a participant information sheet that highlights in layman's terms the study and its risks and benefits?

**Discussion**

 The discussion centres around justifying the use of sham-ECT study in determining the ECT-specific effects rather than placebo or other effects. This is an important research question, which will hopefully be addressed in this study. However, ECT has been used continuously for the treatment of schizophrenia for over 80 years. An absence of evidence through a randomized sham-controlled trial does not necessarily mean that there is *no* evidence for the role of ECT in the treatment of schizophrenia. With this in mind, a major limitation here is an absence of a contrarian discussion on the ethics of sham-ECT trials. A delay of true-ECT treatment for nearly 3 weeks (9 sessions) and subjecting a patient to repeated administration of IV anaesthesia/muscle relaxant without electrical stimulation in 50% of the participants is ethically troublesome. What would be the answer given while obtaining consent when a patient/family asks: 'What is my benefit in participating in the trial if I receive a 'sham- treatment'?'. At the very least, these matters should be acknowledged.

Is the study design appropriate for the research question?

Yes

Is the rationale for, and objectives of, the study clearly described?

Yes

Are sufficient details of the methods provided to allow replication by others?

Yes

Are the datasets clearly presented in a useable and accessible format?

Not applicable

Reviewer Expertise:

Electroconvulsive Therapy.

I confirm that I have read this submission and believe that I have an appropriate level of expertise to confirm that it is of an acceptable scientific standard, however I have significant reservations, as outlined above.
